# Appraising LaQshya’s potential in measuring quality of care for mothers and newborns: a comprehensive review of India’s Labor Room Quality Improvement Initiative

**DOI:** 10.1186/s12884-024-06450-x

**Published:** 2024-04-04

**Authors:** Shalini Singh, Zabir Hasan, Deepika Sharma, Amarpreet Kaur, Deeksha Khurana, J N Shrivastava, Shivam Gupta

**Affiliations:** 1 Department of International Health, Johns Hopkins Bloomberg School of Public Health, Baltimore, USA; 2 BRAC James P. Grant School of Public Health, BRAC University, Dhaka, Bangladesh; 3 National Health Systems Resource Center, New Delhi, India; 4 Department of Epidemiology, Biostatistics and Informatics, Perelman School of Medicine, University of Pennsylvania, Philadelphia, USA; 5 Johns Hopkins India Pvt Limited, New Delhi, India; 6 The Global Fund, Geneva, Switzerland

**Keywords:** Maternal and newborn care, Intrapartum care, Quality improvement, Quality of care assessment, Maternal and newborn care assessment

## Abstract

**Background:**

Poor intrapartum care in India contributes to high maternal and newborn mortality. India’s Labor Room Quality Improvement Initiative (LaQshya) launched in 2017, aims to improve intrapartum care by minimizing complications, enforcing protocols, and promoting respectful maternity care (RMC). However, limited studies pose a challenge to fully examine its potential to assess quality of maternal and newborn care. This study aims to bridge this knowledge gap and reviews LaQshya’s ability to assess maternal and newborn care quality. Findings will guide modifications for enhancing LaQshya’s effectiveness.

**Methods:**

We reviewed LaQshya’s ability to assess the quality of care through a two-step approach: a comprehensive descriptive analysis using document reviews to highlight program attributes, enablers, and challenges affecting LaQshya’s quality assessment capability, and a comparison of its measurement parameters with the 352 quality measures outlined in the WHO Standards for Maternal and Newborn Care. Comparing LaQshya with WHO standards offers insights into how its measurement criteria align with global standards for assessing maternity and newborn care quality.

**Results:**

LaQshya utilizes several proven catalysts to enhance and measure quality- institutional structures, empirical measures, external validation, certification, and performance incentives for high-quality care. The program also embodies contemporary methods like quality circles, rapid improvement cycles, ongoing facility training, and plan-do-check, and act (PDCA) strategies for sustained quality enhancement. Key drivers of LaQshya’s assessment are- leadership, staff mentoring, digital infrastructure and stakeholder engagement from certified facilities. However, governance issues, understaffing, unclear directives, competency gaps, staff reluctance towards new quality improvement approaches inhibit the program, and its capacity to enhance quality of care. LaQshya addresses 76% of WHO’s 352 quality measures for maternal and newborn care but lacks comprehensive assessment of crucial elements: harmful labor practices, mistreatment of mothers or newborns, childbirth support, and effective clinical leadership and supervision.

**Conclusion:**

LaQshya is a powerful model for evaluating quality of care, surpassing other global assessment tools. To achieve its maximum potential, we suggest strengthening district governance structures and offering tailored training programs for RMC and other new quality processes. Furthermore, expanding its quality measurement metrics to effectively assess provider accountability, patient outcomes, rights, staff supervision, and health facility leadership will increase its ability to assess quality improvements.

**Supplementary Information:**

The online version contains supplementary material available at 10.1186/s12884-024-06450-x.

## Background

India has made significant progress in reducing maternal and new-born mortality since 2005. The health systems reforms implemented nationwide as part of the National Health Mission have averted millions of new-born deaths and saved the lives of thousands of women [1, 2]. Despite this declining trend, the burden of maternal and neonatal mortality remains high. The current Maternal Mortality Ratio (MMR) is 97 per 100,000 live births, and the neonatal mortality rate (NMR) is 20 per 1000 live births [3, 4]. Almost half the maternal deaths, 40% of all stillbirths, and neonatal deaths occur during labor, on the day of birth [5]. This happens despite having a roster of proven interventions and technologies that can effectively address the causes of perinatal mortality [6–8].

While the inequitable arrangements of service delivery and inefficient health systems can be considered the root cause, poor quality of care is one of the significant contributors to the excess mortality among mothers and children [9–11]. “*A lack of quality care in the health facilities of India is perceived as the factor most contributing to the maternal deaths by family members of deceased women*” [12]. Disrespectful treatment during childbirth is also widespread, with 70% of women reporting experiencing some form of mistreatment [13]. To develop effective solutions, better measurements of healthcare quality are needed to pinpoint where interventions would have the greatest impact to address both systemic and care delivery issues to improve maternal and child health outcomes.

### Assessment of maternal and new-born services and quality of care in the global context and India

Globally, several standardized facility assessment tools exist to comprehensively assess maternal, newborn and child health. However, their objective and structure vary significantly. As example – the Service Delivery Indicators (SDI) initiative of the World Bank aims to provide benchmarking of service standard [14], Service Availability and Readiness Assessment (SARA) developed by the World Health Organization (WHO) monitors indicators related to the continuum of care [15], and lastly Service Provision Assessment (SPA) spearheaded by Demographic and Health Survey Program (DHS) includes several quality of care indicators – related to antenatal care, family planning, and sick child care – but not related to prenatal care during childbirth [16]. Recently, WHO led the development of the Harmonized Health Facility Assessment (HHFA) through a collaborative, multi-stakeholder process. The HHFA provides modules and tools for a comprehensive, standardized assessment of health facility services, including quality of care through record reviews, to generate evidence to strengthen health systems [17]. However, prominent assessment methods specifically for the quality of maternity and newborn care in health facilities used globally include WHO’s Standards for enhancing the quality of maternal and newborn care [18], a toolkit developed by JHPIEGO for site assessment and strengthening of maternal and newborn health programs [19], and the Maternal and Child Health Integrated Program Health Facility Survey Toolkit by USAID [20]. Among these, the WHO framework is widely used and considered the only unified global tool with a comprehensive set of indicators for maternal and newborn health [21].

In India, women can access maternal health services at different levels, ranging from community to the highest tier of healthcare facilities, encompassing both the public and private health systems. In the public health system, Sub-Centres Health and Wellness Centre (SC-HWCs) at 3000–5000 population, serve as the primary level facilities for basic maternal health services, managed by a trained Community Health Officer, at least one or two female multi-purpose workers and 4–5 ASHAs (Community Health Workers) who conduct outreach services for pregnancy registration, antenatal and postnatal care, and family planning. Primary Health Centres-HWC (PHC-HWC) at 20,000–30,000 in rural and for minimum 50,000 population in urban areas act as women’s initial point of contact with physicians, offering more expanded services and with a longer time or 24-hour availability of services.

The first tier of secondary care facilities is 50–100 bedded Community Health Centres (CHCs) at the sub-district level. CHCs have specialists in obstetrics, gynaecology, paediatrics, and anaesthesia, along with trained nursing staff. CHC’s offer 24-hour delivery, referrals for complications, postnatal care for 0 & 3rd day, management of obstetric complications and Basic Emergency Obstetric Care. CHCs designated as first referral units (FRUs) can perform Caesarean sections and have blood storage units. Sub-divisional (100 bedded) at sub-districts and District hospitals (50–500 bedded) are other secondary facilities, that provide normal delivery care, Caesarean sections, manage complicated deliveries that CHCs cannot handle, address associated maternal and neonatal complications. SDHs are expected to have 2 specialists each in obstetrics and gynecology, pediatrics, and anesthesia, along with five medical officers and adequately trained nursing staff to ensure the provision of comprehensive maternity care services. While District Hospitals have larger number of specialists [2–6] for obstetrics and gynecology, pediatrics, and anesthesia, an adequate number of medical officers and trained nursing staff to deliver comprehensive maternity care services. Government medical colleges and specialty hospitals, tertiary facilities, handle the most complex maternal and neonatal health conditions [22–25].

Over the last two decades, several programs have been implemented to improve the quality of care in these health facilities [26]. The Ministry of Health and Family Welfare (MoHFW) has been implementing the National Quality Assurance Program (NQAP) as the primary means of quality assessment of health programs since 2013. As part of the NQAP, MoHFW has been using National Quality Assurance Standards (NQAS), accredited by the International Society for Quality in Health Care (ISQUA), which further adapted measures based on the three aspects of the Donabedian Model of Quality of Care– the Structure, Process, and the Outcome [27]. Nonetheless, India’s record of ensuring high-quality maternal and newborn care remained suboptimal [11, 28, 29]. Several factors contribute to suboptimal care in health facilities, including delays in providing care to intrapartum mothers, incomplete adherence to safe birth protocols, a lack of organized preparation for birth, referrals not resulting in treatment, low competence among staff to manage obstetric complications, the absence of skilled birth attendants, and instances of staff abuse and neglect during delivery [30–33]. This underscored the need to implement a robust facility-level quality assessment program specifically targeting maternity and newborn care to improve health outcomes.

In response, the government launched the Labor Room Quality Improvement Initiative (LaQshya) in 2017. LaQshya aims to reduce clinical complications and improve outcomes by enhancing the quality of maternal and newborn care. Its goals include decreasing complications such as hemorrhage, retained placenta, preterm birth, preeclampsia, obstructed labor, sepsis, and asphyxia, etc. It also focuses on building capacities for prompt stabilization of above complications, timely referrals, and building an effective two-way follow-up system through effective communication between health providers at different levels of the health care system. Extending respectful maternity care (RMC) to all pregnant women is another critical objective of LaQshya [34]. During its conceptualization, measurement metrics, and processes to assess the quality of intrapartum and immediate postpartum care in LaQshya were drawn from NQAS.

Over the last six years since its launch, only a few studies have been conducted to examine LaQshya’s performance, which indicated structural and process related improvements in service delivery under LaQshya, including infrastructure upgrades, new protocols, training programs, and infection control practices [35, 36].

However, these studies are limited in scope and depth, providing insufficient insights into the overall effectiveness of LaQshya. Either they focus on a few aspects of the program, such as RMC or adherence to guidelines, or they report on changes experienced due to the program from a single health facility. The studies lack a thorough examination of LaQshya’s implementation experience that can help in identifying bottlenecks faced with the program and specific areas of improvement to strengthen the program.

Furthermore, to date, no comparative analysis has been conducted to evaluate LaQshya against global standards, such as the framework outlined by the WHO [18]. Such an analysis could provide valuable insights into how LaQshya aligns with established international benchmarks for maternal and newborn healthcare.

This paper aims to address these knowledge gaps and appraise LaQshya’s potential in measuring the quality of care for mothers and newborns. We begin by offering a descriptive case analysis of LaQshya’s operational elements, strengths, implementation experience, and challenges. Next, we compare LaQshya’s measurement metrics and facility assessment tools with the WHO’s Standards for Improving Quality of Maternity and Newborn Care in health facilities. Through the descriptive case analysis and comparative assessment, we draw insights into LaQshya’s capacity to measure the quality of intrapartum care for mothers and newborns in public health facilities.

## Methods

We implemented a “Descriptive Case Design” to review the structure of LaQshya and compare it with WHO’s Standards for Improving Quality of Maternity and Newborn Care. There are inherent challenges for appraising any new quality improvement programs like LaQshya due to the contextual dependencies and interconnected activities that are implemented at varying times and scales within the health systems, in addition to the lack of standardized evaluation frameworks [37]. A descriptive case design is the most relevant method in this context for an in-depth, detailed examination of the components, implementation strategies, strengths, and weaknesses of the program rather than the program outcomes. Furthermore, comparing and contrasting with the WHO standards through a descriptive case design will provide a rich, contextual understanding of similarities and differences between the two quality improvement frameworks [38].

**Fig. 1:**
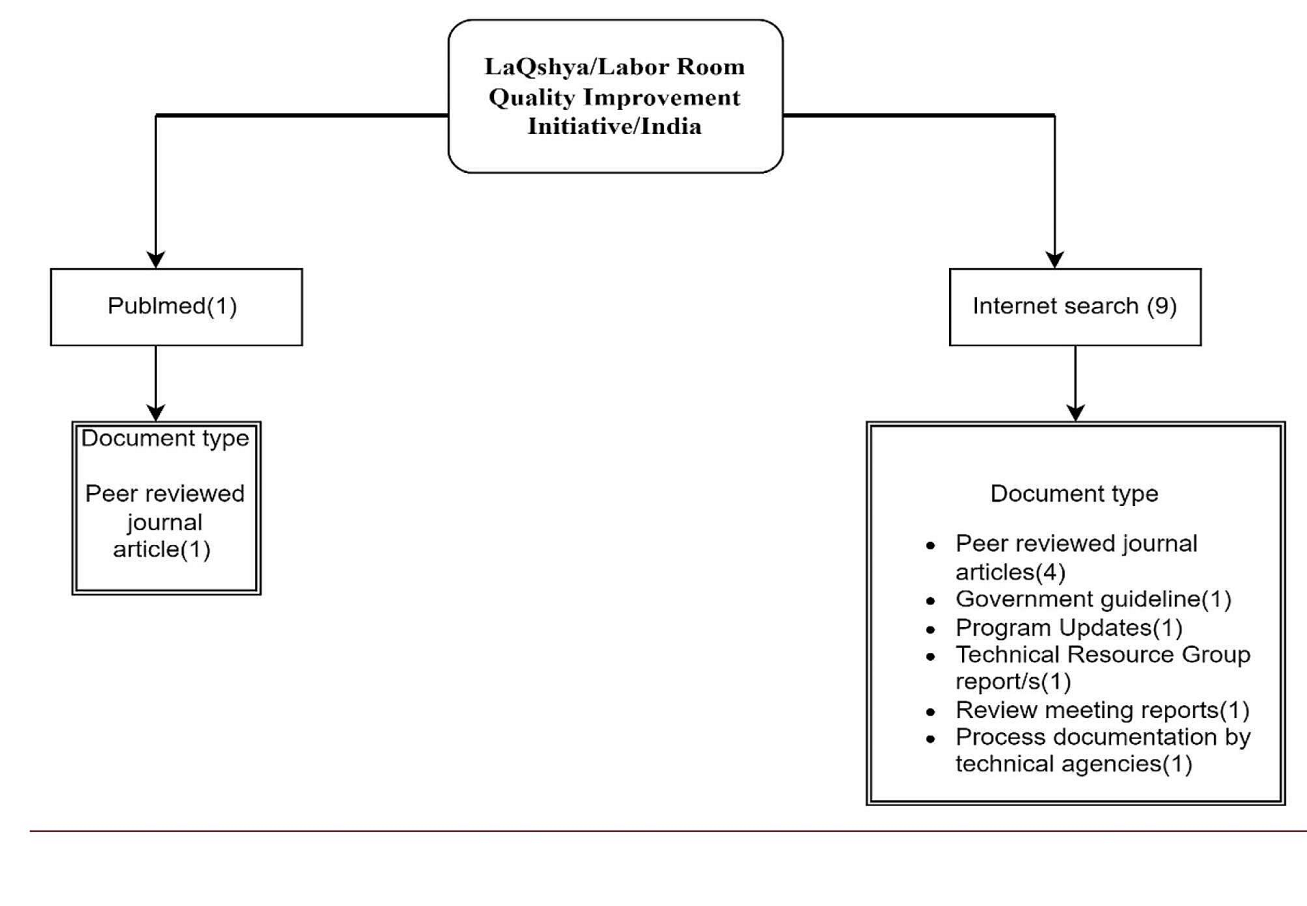
Types of documents reviewed

In our analytical approach, we first conducted a document review to obtain information and insights about LaQshya’s strategy, operational plan, and implementation experience. A combination of searches on the Internet and PubMed were used to collect relevant materials. PubMed searches were conducted using the following search query “LaQshya“[All Fields] AND ((((“Maternal Health“[MeSH Terms] OR “Maternal Health“[All Fields] OR “Infant Health“[MeSH Terms] OR “Infant Health“[All Fields]) AND “Or“[All Fields]) AND “Neonatal Health“[All Fields]) OR “labor, obstetric“[MeSH Terms] OR “labour“[All Fields]) AND (“quality improvement“[MeSH Terms] OR “quality assurance, health care“[MeSH Terms] OR “quality improvement*“[All Fields] OR “Quality Assurance“[All Fields] OR “Quality Monitoring“[All Fields]) AND (“India“[MeSH Terms] OR “India“[All Fields]) AND 2017/01/01:2024/02/29[Date - Publication] AND “english“[Language]. As LaQshya program was launched in 2017 we considered articles published after 2017. Only one study was found pertaining to the program and was included in the documents review (Supplementary File 1 for detailed search strategy). An aadditional nine documents including LaQshya-related published peer-reviewed articles, and other grey literature - such as program guidelines, program updates, process documentation, technical resource group reports, and meeting reports - were obtained through internet searches using key terms LaQshya Program, LaQshya Initiative India, Labor Room Quality Improvement Initiative in India. We used the identified set of ten documents and conducted a detailed content review (See Fig. 1 for distribution of documents used).

A data charting form was used to extract the evidence from the documents for thematic exploration to prepare the descriptive case analysis (Supplementary File 2).

Next, we compared the LaQshya quality measurement system with the WHO’s Standards [18]. The WHO standards adopt a health systems framework and include eight standards of quality of care to assess, improve, and monitor services being received by pregnant women and newborns during childbirth in health facilities. Each standard embodies a certain number of quality statements, which are further linked with specific indicators or measures. The eight standards provide a clear and broad outline of the necessary requirements to attain high-quality care during childbirth, and quality statements linked to each standard were formulated to drive measurable improvements in the quality of care. The measures were established as the actual criteria used to assess and monitor the quality of care linked to each specified in the quality statements [18]. There are a total of 352 indicators or measures, which include 164 input, 110 output/process, and 78 outcome measures [18]. These standards, quality statements, and measures have been referred to widely in the LMIC settings, used as guidelines to understand quality gaps [31, 32], and used to assess the ability of existing tools to optimally capture quality of care indicators [33]. We believed that comparing LaQshya against the WHO standards would provide valuable insights into how LaQshya’s measurement criteria compare with established global standards to assess the quality of maternity and new-born care. For additional information on the WHO’s Standards, please review https://www.who.int/publications/i/item/9789241511216.

The LaQshya quality measurement system drawn from NQAS includes slightly different elements which are structured into areas of concern, standards, measurable elements, and checkpoints. These elements are linked to two different checklists to assess the quality of care in labour rooms and maternity operation theatres (OT), respectively. Each of these checklists includes eight areas of concern – inputs, service provision, support services, patient rights, clinical services, quality measurement systems, infection control, and outcomes [34]. Combining both the labour room and maternity OT checklists, LaQshya embodies- a total of 58 standards, 181 measurable elements, and 429 checkpoints to monitor the eight areas of concern. Among these, 8 standards, 50 measurable elements, and 269 checkpoints are unique to either of the checklists, with the remaining elements common to both. Like the WHO’s framework, standards in LaQshya are also “statement of requirements of a particular aspect of quality” and measurable elements are specific attributes of a standard that need to be reviewed for assessing the adherence to a particular standard. Lastly, checkpoints are the tangible elements that can be recorded, scored, and objectively observed [39], which is very similar to the WHO measures.

We adapted the method presented by Brizuela and colleagues [40] as our analytical approach, where the authors equated quality standards proposed by WHO with other facility-level assessment tools [40].

Our comparative analysis specifically focused on measures from WHO standards and measureable elements/checkpoints in LaQshya’s framework. We initially developed a comparison matrix in Excel with WHO standards, the corresponding quality statements, and measures. Utilizing the keywords from WHO measures, we conducted a comprehensive search for measurable elements and checkpoints in the LaQshya checklists for labour room and maternity OTs [41]. The matching measurable elements and checkpoints were then added to the matrix against the WHO measure being compared. We labeled the WHO measures for which no matching results were found in the LaQshya checklists as “Not covered” in the comparison matrix (Supplementary File 3). Once all WHO measures were thoroughly assessed to determine their coverage, a score of 1 was assigned to each measure covered, and 0 for those Not covered. Using descriptive statistics, we calculated how many quality measures could be assessed using the LaQshya checklist.

## Results

The study’s results are presented in three sections. Part A details the Organization of LaQshya, focusing on its strategy, enablers, and innovations. Part B provides a summary of LaQshya’s comparative assessment with WHO standards, and Part C reports on LaQshya’s implementation experience, program progress, and challenges.

### Organization of LaQshya

#### Strategy

**Fig. 2:**
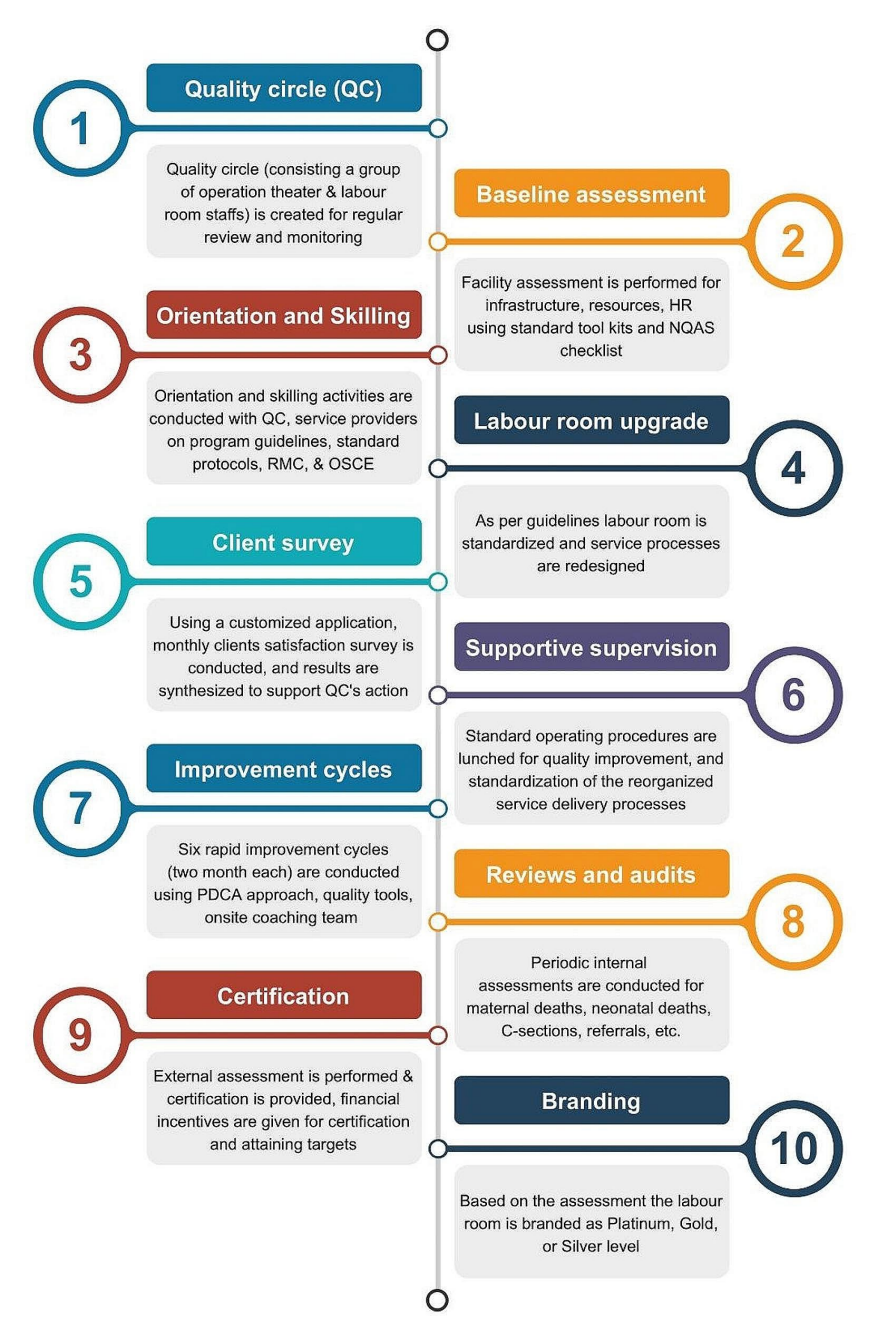
Ten Steps towards LaQshya Certification

LaQshya adopts a multipronged approach to improve the quality of intrapartum and immediate post-natal care for mothers and newborns in government-funded (public) health facilities. It targets three key strategic levers- (a) remodeled and standardized labor rooms (LRs) and maternity operation theatres (OTs) (b) protocol-based care around childbirth, and (c) enhanced client satisfaction through “respectful maternity care” (RMC). It is a voluntary program for the health facilities to participate. And once registered, the facilities must follow ten sequential steps to get certified (Fig. 2).

The LaQshya program is implemented as a “facilitated process”, wherein both central and state governments provide additional resources to aid the assessment and accreditation process. After registration, health facilities are required to submit their action plan, the government provides extra funding, workforce, and resources to support the attainment of LaQshya’s quality of care enhancements. LaQshya’s mentoring structure comprises program officers, representatives from medical colleges, development partners, quality of care experts, nurse mentors, etc. The groups enhance the program support by preparing implementation plans, offering on-site support, conducting training, identifying best practices and innovations, and overseeing maternal and infant death surveillance along with Quality Circles (QC) monitoring. The QC is the facility-level structure that executes all LaQshya interventions, clinical protocols, and tools in the labor room and maternity OT with support from the hospital quality team. These QC use standard quality improvement methodology: incorporate six Rapid Improvement Cycles (thematic campaigns implemented every two months for phased learning), evaluation, feedback, and mastering quality protocols for sustainability. QC records gaps related to the selected theme, and for process improvement, use Plan – DO – Check –Act (PDCA) cycles.

**Fig. 3:**
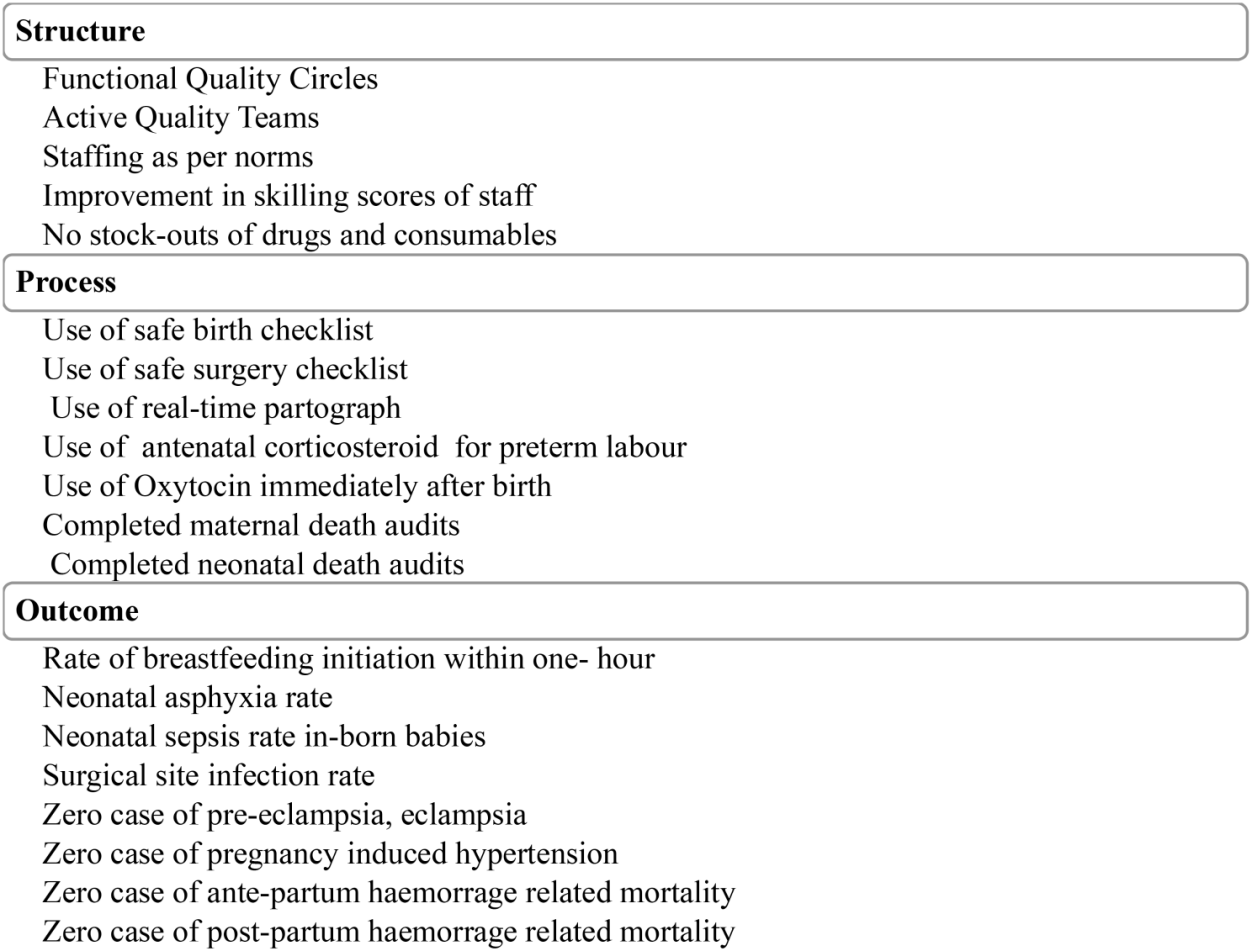
Indicators for health facility performance measurement and incentive disbursal after LaQshya certification

The full suite of LaQshya interventions is to be implemented over a period of 18 months across four different phases - preparatory, assessment, improvement, and evaluation. At the end of 18 months quality of care evaluation of labor room and maternity OTs is undertaken by external assessors. At the national and sub-national, the government has been developing extensive programatic and institutional structures to support health facilities to achieve LaQshya certification. The existing Quality Assurance committees established at national and sub-national levels for the NQAS are supporting LaQshya-specific mentoring activities. This also includes experts empaneled by the National Health Systems Resource Centre - the Technical Secretariat for the LaQshya program at the national level. Assessors review the quality of care as per the standard quality measurement tool - the LaQshya checklists [41]. State and national level teams of quality assessors’ complete assessment for accreditation in two rounds, respectively, and certify the health facility. Thereafter, performance is measured on a set of 20 pre-specified structure, process, and outcome measures listed in Fig. 3 [34].

The information generated through the quality improvement process enables performance tracking to disburse incentives to health facilities. Incentives are released on achievement of quality certification of labor room and/or OT, attainment of at least 75% of commensurate facility level targets for indicators listed in Fig. 2, its verification by the State Quality Assurance Committee, and on achieving 80% of the beneficiary satisfaction rate.

#### Enablers and innovations

From our review, we identified four key drivers for LaQshya’s progress, particularly observed in the states that demonstrated good progress. First, effective leadership and commitment across all levels drive change [34]. Second, proactive and comprehensive actions to address infrastructure, human resources, and quality of care gaps, as observed in MP and Gujarat, are critical to advancing the program. Next, systems of weekly progress reviews and on-the-job training of clinical providers by master trainers, exemplified in Chandigarh, are necessary. Lastly, proactive collaboration and stakeholder engagement through experience sharing by certified facilities, seen in Tamil Nadu, helps in overcoming challenges (such as staff reluctance), towards the implementation of extensive and new program activities under LaQshya [42].

During implementation of LaQshya, several digital innovations for training, program monitoring and certification process have also evolved. SaQsham (Strengthening Quality and Safety of Health Facility Assessments), a web platform, has been developed to document and review all the tasks related to LaQshya certification in the country. This is expected to support the program managers in the intensive certification process, through systematically organizing the exhaustive information required for the assessment process. This platform also assists in data storage for tracking quality improvement changes, maintains transparency, reduces variability, ensures time efficiency, and acts as a digital backup system for the data [43].

In recent years, additional digital tools have been developed in collaboration with development partners to support implementation of LaQshya [44]. For example, a mobile Integrated Safe Delivery App is being used to enhance healthcare providers’ skills in safe delivery and newborn care through self-learning. Various program monitoring tools, including offline scorecards, action plan templates, LaQshya MIS/dashboards, and online outcome indicators tools, have been developed. Mera Aspatal, a Ministry of Health client satisfaction tool, has been adapted to gather client perceptions on care in labor rooms and maternity OTs [44]. LaQshya assessments had to be re-oriented due to COVID-19 restrictions, and virtual platforms for training, mentoring, and assessments served as key enablers to maintain program continuity.

### Comparative analysis of Laqshya with the WHO standards

**Fig. 4:**
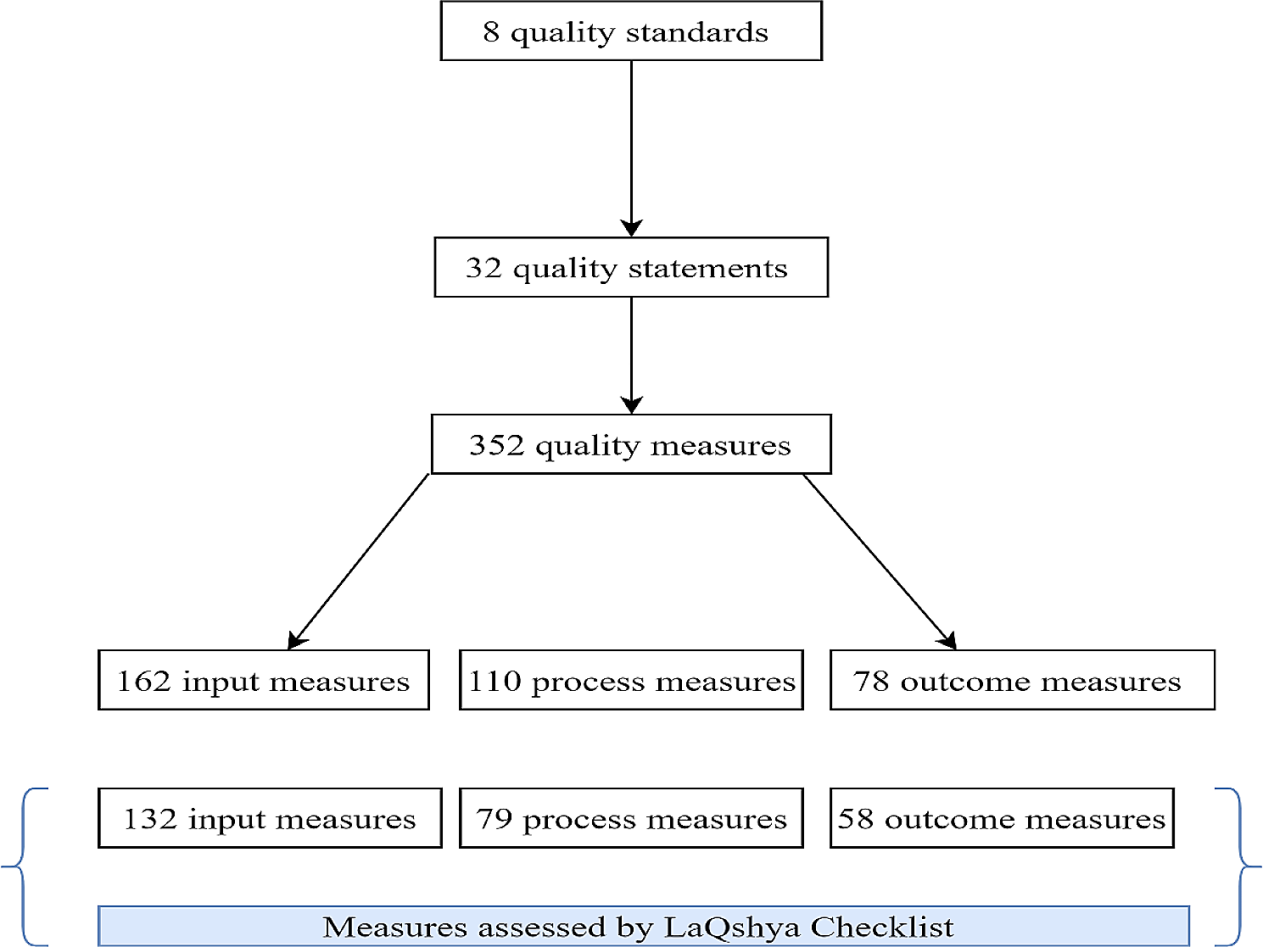
WHO measures under review and assessed by LaQshya checklists

Our comparative analysis reveals that out of the 352 WHO quality measures, the LaQshya checklists cover and assess 269 (76%) measures. Figure 4 depicts the number of WHO standards, statements, and measures under review and measures assessed by the LaQshya checklists.

The extent of overlap in the LaQshya checklist is highest for input measures (80%), followed by outcome measures (74%) and is lowest for process measures (72%).

**Fig. 5:**
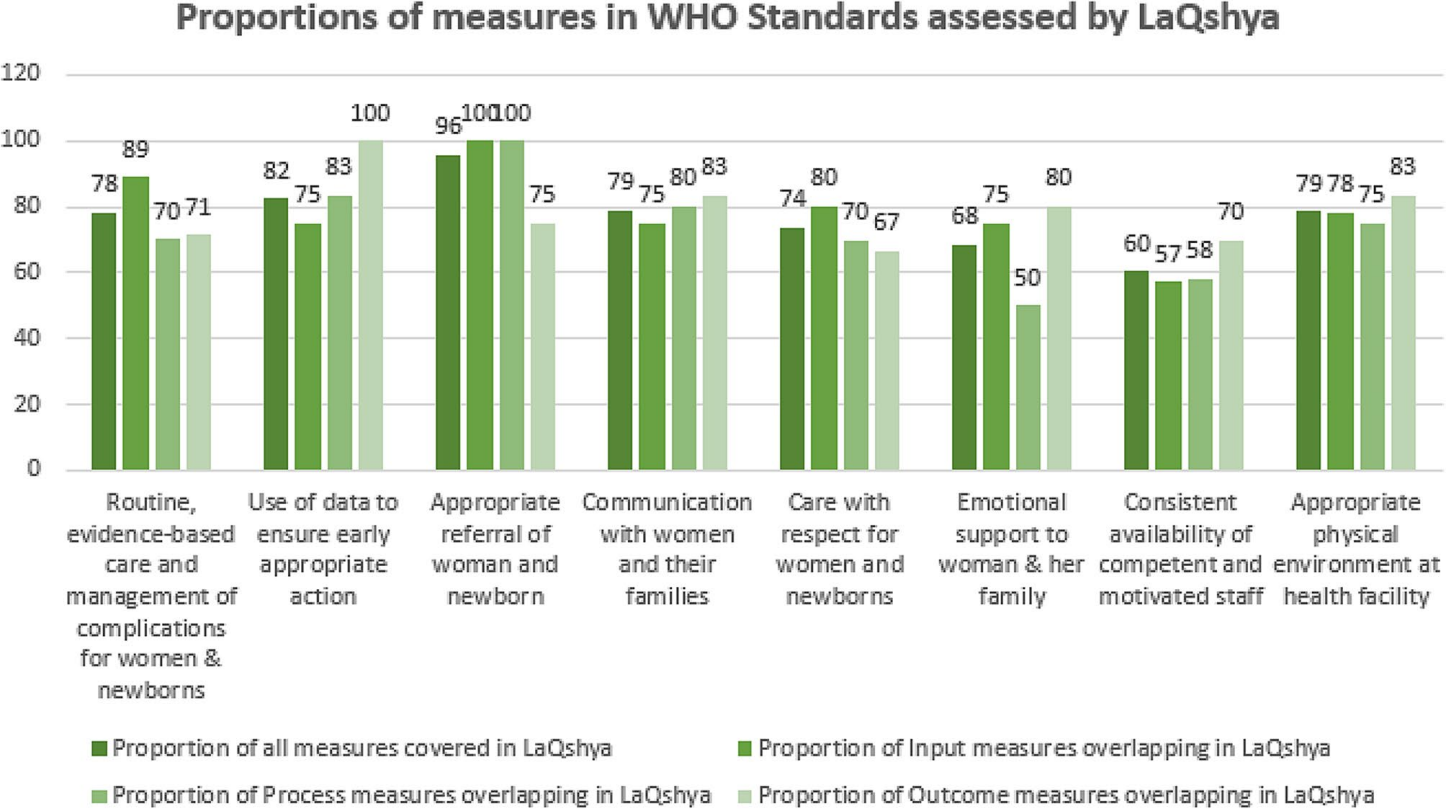
Standard wise proportion of measures in WHO standards assessed by LaQshya

There is significant variation observed in the extent to which LaQshya checklists can measure each of the eight WHO standards (Figure: 5) While all standards are partially assessed, the tool excels in evaluating standard 3, pertaining to referrals for conditions that cannot be adequately addressed using the available resources, coverage of up to 96% WHO quality measures.

It also has a moderate capacity (about 74–82%) to assess evidence-based care and management of complications (standard 1), use of health information system (standard 2), effective communication (standard 4), women and newborns receiving care with respect and dignity (standard 5).and appropriate physical environment, medicines, supplies (standard 8). A relatively lower capacity (60–68%) is observed to measure provision of emotional support to mothers and families (standard 6) and availability of competent, motivated staff (standard 7).

Table 1 summarizes the ability of LaQshya checklist to measure each of the 32 WHO quality statements. Columns report proportion of input, process and outcome measures within the quality statement that are captured by the LaQshya. Albeit partially, LaQshya checklist can assess all quality statements from the WHO framework. It assesses all 100% measures for seven quality statements (1.2, 3.1,3.3, 4.2,5.1, 6.1, 7.1). For 16 quality statements (1.1a/b, 1.3, 1.4, 1.5, 1.6a/b, 1.7b, 1.8, 2.1, 2.2, 3.1, 3.2, 5.3, 8.1 to 8.3), LaQshya can capture a significant subset (more than 70%) of the quality measures. The quality statements which have 60% or less coverage in LaQshya are those related to- prevention of unnecessary or harmful practices during labor and postnatal period (1.9); elimination of mistreatment towards women and newborns (5.2); support for women during childbirth (6.2) and availability of competent skilled birth attendants and support staff (7.2) and presence of managerial and clinical leadership to foster an environment that supports facility staff in continuous quality improvement.

**Table 1:** Performance of LaQshya checklist under review according to WHO quality statements

WHO Quality Statement	WHO Quality Statement	Input (%)	Process (%)	Outcome (%)	Overall (%)
1.1a	Women are assessed routinely on admission and during labour and childbirth and are given timely, appropriate care.	100	60	100	83
1.1b	Newborns receive routine care immediately after birth.	100	80	100	92
1.1c	Mothers and newborns receive routine postnatal care.	63	56	100	63
1.2	Women with pre-eclampsia or eclampsia promptly receive appropriate interventions, according to WHO guidelines.	100	100	100	100
1.3	Women with postpartum haemorrhage promptly receive appropriate interventions, according to WHO guidelines.	100	50	60	73
1.4	Women with delay in labour or whose labour is obstructed receive appropriate interventions, according to WHO guidelines.	100	86	50	81
1.5	Newborns who are not breathing spontaneously receive appropriate stimulation and resuscitation with a bag-and-mask within 1 min of birth, according to WHO guidelines.	100	100	50	89
1.6a	Women in preterm labour receive appropriate interventions for both themselves and their babies, according to WHO guidelines.	75	100	100	90
1.6b	Preterm and small babies receive appropriate care, according to WHO guidelines.	100	100	50	82
1.7a	Women with or at risk for infection during labour, childbirth or the early postnatal period promptly receive appropriate interventions, according to WHO guidelines.	100	80	0	70
1.7b	Newborns with suspected infection or risk factors for infection are promptly given antibiotic treatment, according to WHO guidelines.	100	0	100	78
1.8	All women and newborns receive care according to standard precautions for preventing hospital-acquired infections.	100	75	67	86
1.9	No woman or newborn is subjected to unnecessary or harmful practices during labour, childbirth and the early postnatal period.	50	43	NA	46
2.1	Every woman and newborn has a complete, accurate, standardized medical record during labour, childbirth and the early postnatal period.	33	100	NA	67
2.2	Every health facility has a mechanism for data collection, analysis and feedback as part of its activities for monitoring and improving performance around the time of childbirth.	100	67	100	91
3.1	Every woman and newborn is appropriately assessed on admission, during labour and in the early postnatal period to determine whether referral is required, and the decision to refer is made without delay.	100	100	100	100
3.2	For every woman and newborn who requires referral, the referral follows a pre-established plan that can be implemented without delay at any time.	100	100	67	90
3.3	For every woman and newborn referred within or between health facilities, there is appropriate information exchange and feedback to relevant health care staff.	100	100	NA	100
4.1	All women and their families receive information about the care and have effective interactions with staff.	50	67	75	64
4.2	All women and their families experience coordinated care, with clear, accurate information exchange between relevant health and social care professionals	100	100	100	100
5.1	All women and newborns have privacy around the time of labour and childbirth, and their confidentiality is respected.	100	100	100	100
5.2	No woman or newborn is subjected to mistreatment, such as physical, sexual or verbal abuse, discrimination, neglect, detainment, extortion or denial of services	63	60	33	56
5.3	All women have informed choices in the services they receive, and the reasons for interventions or outcomes are clearly explained.	100	67	67	80
6.1	Every woman is offered the option to experience labour and childbirth with the companion of her choice.	100	100	100	100
6.2	Every woman receives support to strengthen her capability during childbirth.	50	25	75	50
7.1	Every woman and child has access at all times to at least one skilled birth attendant and support staff for routine care and management of complications.	100	100	100	100
7.2	The skilled birth attendants and support staff have appropriate competence and skills mix to meet the requirements of labour, childbirth, and the early postnatal period.	33	43	40	39
7.3	Every health facility has managerial and clinical leadership that is collectively responsible for developing and implementing appropriate policies and fosters an environment that supports facility staff in continuous quality improvement.	50	50	100	57
8.1	Water, energy, sanitation, hand hygiene and waste disposal facilities are functional, reliable, safe and sufficient to meet the needs of staff, women and their families.	64	NA	100	71
8.2	Areas for labour, childbirth and postnatal care are designed, organized and maintained so that every woman and newborn can be cared for according to their needs in private, to facilitate the continuity of care.	88	100	100	90
8.3	An adequate stock of medicines, supplies and equipment is available for routine care and management of complications.	85	67	50	78

*NA = For these variables, the denominator is 0, there was no input/output/outcome listed in the WHO Standards for the Quality Statements

In absolute terms, out of 83 (24%) unassessed quality measures in LaQshya, the highest number [37] is from WHO Standard 1, focusing on care during labor, childbirth, and postnatal period. Standard 7, dealing with available staff for care, follows with 22 measures. The remaining standards have 3 to 13 missing measures in LaQshya (See Supplementary File 4 for details).

LaQshya falls short in assessing important input measures, such as those related to the supportive supervision of the health facility staff for- evidence based care, communication with mother and families, leadership, and management skills. Critical inputs specified by WHO to assess standard 5 on accountability mechanisms and protocols to ensure care of mothers with respect and dignity are also missing.

Amongst the WHO process measures, LaQshya includes all for physical environment and supplies (standard 8), but it lacks multiple process assessments for evidence-based care (standard 1). These omissions span various aspects such as providing women pain relief options, specific procedures such as adherence to Robson classification for C sections, antibiotic administration for perineal tears, newborn infections etc. Moreover, LaQshya doesn’t cover essential parameters like protocols of newborn referrals, staff communication, grievance redressal, women’s rights, and measures to review competence, mentoring and supervision of staff to support quality improvement activities.

Several WHO outcome measures such as those assessing- management of complications during labour/ childbirth and the early postnatal period and effective communication by providers with women and families are missing in LaQshya. Additionally, it doesn’t incorporate patient-reported outcomes, respectful treatment, patient rights, and staff satisfaction.

### Implementation experience

#### Progress

LaQshya has an ambitious target to accredit 2445 high case load tertiary and secondary care public health facilities across India. The program has been initiated across all government medical college hospitals, district hospitals & equivalent health facilities, all designated FRUs and high case load CHCs.

Other than slow progress owing to pandemic-related restrictions in 2020–2021, the number of LaQshya-certified health facilities has been steadily increasing since its launch in 2017 [43]. As of December 2022, about 47% of the targeted health facilities (644 labor rooms and 504 maternity OTs) have achieved national-level certification [45]. A total of 122 labor rooms at Community health Centers, 116 at sub-district hospitals, 360 at district hospitals and 45 at medical colleges are quality certified for LaQshya.504 maternity OTs have been certified for LaQshya, and include 49 at CHCs, 96 at SDH, 317 at district hospitals, and 42 at medical colleges. However, this progress is not uniform across India. State of Madhya Pradesh (MP) has the highest number of certified labor rooms and maternity operation theatres (144, 91LR, 53MOT), closely followed by Maharashtra (143, 72LR, 71MOT), Karnataka (122, 62LR, 60MOT), Gujarat (106, 58LR, 48MOT), Andhra Pradesh (76, 34LR, 43MOT) and Tamil Nadu (76, 38LR, 38MOT). Progress is slow in other states, while, the state of Meghalaya is yet to initiate the certification process under LaQshya [43].

#### Challenges

Our review highlighted various barriers for LaQshya [35, 44, 46, 47]; in both, its implementation within health facilities and program management. Most challenges evolve from issues surrounding governance or interconnected factors of understaffing and limited program support. Frequent changes in administrative leadership and limited availability of both clinical and managerial manpower adversely impact the program. At times, program or hospital leaders lack clarity about LaQshya’s requirements [44, 48], resulting in resource and infrastructure constraints. Flawed prioritization of hospital management also leads to a lack of program clarity, as the focus often remains on the certification process, neglecting the reviews and planning needed to improve clinical protocols crucial for sustained quality improvements.

Most of these challenges can be resolved through supportive supervision and mentoring by institutional structures - the State Mentoring Group (SMGs) or the District Coaching Teams (DCTs) for LaQshya. These were not yet fully functional, at least in the seven states for which process documentation is available [44]. In certain other states, understaffing and staff orientation issues within State and District Quality Assurance Units are seen to be hindering the effective management of LaQshya [48]. Barriers are also observed due to inadequate competencies of clinical providers, a critical prerequisite for high-quality care. Trainings in intrapartum protocols have either not been conducted previously, or there is inadequate support to refresh skills by quality circles, or mentoring team visits, as originally anticipated in LaQshya guidelines [44, 49].

Effectively tracking LaQshya’s quality measures demands robust data collection, synthesis, and analysis. However, multiple documentation needs, inadequate integration of LaQshya data into routine health information systems, and facility managers’ limited familiarity with digital measurement tools hamper data management. Data-related concerns also impede client satisfaction assessment, it’s not fully operationalized as the updated version of the digital application- Mera Aspatal is not being utilized [44].

Implementing new LaQshya-specific processes such as rapid improvement cycles, respectful maternity care, and birth companions for delivery support has been particularly difficult for health facilities [50]. QCs that ensure these processes are not functional everywhere [39], and there is an issue of flawed perceptions and limited buy-in from the providers related to these changes [46]. Quantum of incentives for the health facility is perceived to be low to maintain staff motivation for LaQshya, and there are no mechanisms to reward health providers who demonstrate creativity and innovative change ideas for quality improvement. Finally, there is limited community participation to enable joint accountability and ownership of quality processes.

## Discussion

This descriptive case analysis of the LaQshya program reveals several key insights into its ability to assess the quality of intrapartum care for mothers and newborns in India.

First, LaQshya is envisioned as an integrated and comprehensive strategy combining quality assurance and quality improvement methods. The quality assurance component, with external assessments and certification, enables benchmarking and accountability. The quality improvement aspect, through mentoring support and internal reviews by quality circles, drives continuous enhancement [42, 44]. This dual approach allows LaQshya to overcome the limitations of standalone other Quality Assurance (QA) and Quality Improvement (QI) methods. The assessments make judgments about care quality at a moment in time, while the improvement processes enable internal capability building for sustained improvements [51].

Second, LaQshya operationalizes many recognized enablers that catalyze its capacity for quality enhancements and measurement. The program embodies strong technical expertise, leverages a digital infrastructure, provides performance incentives for providing high quality care, and utilizes quality improvement tools like the PDCA cycles [42, 44, 46]. Leadership and governance mechanisms have also been established in the form of quality assurance committees and mentoring groups. When optimally implemented, as seen in states like Madhya Pradesh, these structures can enable LaQshya’s objectives [42, 44, 52].

Third, LaQshya’s assessment checklists align significantly with WHO standards. The comparative analysis shows its better capacity than other global tools in quality assessment for maternal and newborn care. The checklists encompass a larger percentage (76%) of WHO’s 352 quality measures; This exceeds the coverage of other tools such as the Service Provision Assessment (SPA) designed for the Demographic and Health Surveys program, the Service Availability and Readiness Assessment by WHO, the Needs Assessment of Emergency Obstetric and Newborn Care by the Averting Maternal Death and Disability program at Columbia University, as well as the World Bank’s Service Delivery Indicator (SDI) and Impact Evaluation Toolkit for Results Based Financing in Health, which range from 62% for the SPA to only 12% for the SDI [53].

These promising attributes have allowed Laqshya to earn its recognition from the WHO as one of the leading successes in Southeast Asia’s Maternal and Child Health Programs, establishing it as a notable model with immense potential for assessing quality of care [8].

Finally, we found that specific gaps exist in LaQshya’s ability for comprehensive quality assessments. It does not comprehensively measure quality from the patient’s perspective through assessments of rights, experience, and mistreatment. Critical issues of harmful practices and evidence- based care during labor, outcomes of referrals and clinical leadership capabilities are a few other missing elements. Incorporating dimensions around accountability, effective supervision, and building staff capabilities can strengthen LaQshya. Our analysis also highlights variability in LaQshya’s implementation across Indian states. Many challenges persist due to limitations in leadership prioritization, data systems, provider skills, and community engagement. Addressing these barriers can maximize LaQshya’s potential for quality enhancements [43, 44, 46].

### Policy implications

This study’s findings also shed light on specific concerns that must be addressed to enhance both the implementation of LaQshya and the quality assessment checklists.

The emerging concerns in implementation are interconnected and multifaceted, spanning leadership misperceptions, insufficient staff training, clinical competency gaps, sub-optimal data management, and coordination issues and have been observed in other similar settings [54]. These issues aren’t standalone; they highlight the need for improvements in governance structures supporting LaQshya at the district level. Strengthening these structures, establishing local networks with coaching and quality improvement teams, and utilizing feedback for better district supervision are crucial for driving quality improvement initiatives in LMICs [55, 56] and can significantly bolster LaQshya’s performance. Additionally, promoting inter-facility collaboration [51], shared learning, and scalable practices through quality improvement collaboratives [52] are promising approaches that could be integrated into LaQshya by enhancing the role of district governance structures.

The issue of low adoption of RMC in facilities implementing LaQshya is reported owing to staff resistance and unfavorable attitude of personnel who render care during childbirth. Previous studies suggest that addressing abuse and promoting respect is a complex process that lacks any quick, technical fix to immediately change individual attitudes, improve patient-provider relationships, or challenge deeply rooted societal norms [57]. Other studies within India and other LMICs emphasize that the barriers to providing RMC are multifaceted. These barriers could encompass health system factors, such as the labor ward’s physical infrastructure, staff shortages, resource limitations, motivational issues, hospital policies, and suboptimal working conditions. Barriers could also include health provider factors of health providers personal beliefs, inadequate professional orientation, and their limited collaboration. Additionally, client-related factors involve women’s and their relatives’ attitudes and unmet expectations [7, 13, 58–60]. A deeper exploration to identify stakeholders’ issues with RMC, utilizing those learnings to build transformational training programs and interventions for RMC have been suggested and could be examined for LaQshya too [57].

The findings of this review indicate that the deficiencies in the LaQshya assessment checklists for assessing the quality of care align to a significant extent with the implementation or program level challenges discussed above. For example, LaQshya fails to measure all critical issues of harmful practices and evidence- based care during labor, mistreatment of mothers or newborns during childbirth, support for women during childbirth, and effective managerial and clinical leadership for continuous quality improvement.

Lack of all measures to assess management of high-risk cases and birth complications in mothers and newborns is a concern. This could possibly limit LaQshya’s ability in attaining its objectives of reducing maternal and neonatal deaths on account of poor quality of care in health facilities. It also does not include performance measures to assess leadership, governance, and staff motivation. Evaluating leadership’s role in quality improvement becomes more critical given the leadership and governance barriers are already restraining LaQshya’s progress in many states. There is close interaction between leadership and organization culture and senior leadership would be vital for integrating innovations like LaQshya into an organization’s vision and operations [54]. They provide direction, allocate resources, manage processes including hospital staff capacities, and cultivate a performance-driven environment for high performance in quality initiatives [61].

LaQshya’s weaker capacity to assess standard 5 on women and newborns receiving care with respect and dignity and provision of emotional support to mothers and families (standard 6) would also need to be addressed more comprehensively if the program objectives of more woman-centered, respectful maternity health-care services are to be achieved in India.

## Limitation

The key limitations of this analysis stem from the reliance on secondary documents, the lack of primary data collection, and the snapshot nature of the assessment. While the analysis would have been enriched by visits to facilities implementing LaQshya or interviews with frontline health workers, we tried to mitigate this by undertaking an extensive review of all available program documents, reports, and scholarly literature. The comparison with WHO standards was also systematically conducted using published tools to ensure standardized and credible benchmarking. Additionally, while the analysis provides insights into a particular time, the focus on measurement frameworks and implementation structures evaluates the overarching capacity and design of LaQshya. The knowledge generated through document review and expert analysis still offers valuable insights into LaQshya’s strengths and weaknesses as a quality measurement tool. The recommendations can inform enhancements in LaQshya’s metrics, governance, and implementation support. While primary data would have added nuance, the analysis provides a robust initial assessment to highlight areas for improving LaQshya’s ability to measure and improve the quality of maternal and newborn healthcare.

We used the WHO framework in entirety for our analysis even though LaQshya is focused only on services provided in labor room and maternity OTs. This is because segregating measures from WHO framework for labor room and maternal OTs was not possible and LaQshya checklists itself had some measures to assess care beyond intrapartum period.

While this review provides valuable insights into LaQshya’s capacity to assess quality of care, it is insufficient to make a conclusive judgment regarding the program’s overall effectiveness, the limited existing studies assessing program outcomes yield mixed results [36, 62, 63]. Only one study from the state of Tamil Nadu highlight improvements in infrastructure, human resources, equipment, supply chain, processes, and outcomes in maternity care on account of LaQshya implementation. These enhancements also led to significant reductions in adverse events, improvements in breastfeeding rates, and reductions in maternal and neonatal complications, ultimately enhancing the quality of care [36]. Additional research is needed to comprehensively grasp the effects of LaQshya on improving the quality of care and its influence on maternal and newborn health outcomes.

## Conclusion

In summary, the comprehensive and innovative design of LaQshya positions it as a powerful model for evaluating the quality of care, surpassing other global assessment tools. To fully harness its potential, we propose three specific actions. Firstly, strengthen the governance structures at the district level to enhance program leadership, mentoring, and supportive supervision for the quality of care. Secondly, gather input from clinical providers to plan transformative training, support, and tailored interventions for RMC and other new quality improvement processes such as Rapid Improvement Cycles and Quality Circles. Finally, expand the range of metrics used to evaluate provider accountability, patient-reported outcomes, RMC, patient rights, staff supervision, and leadership in health facilities.

### Electronic supplementary material

Below is the link to the electronic supplementary material.

Supplementary Material 1

Supplementary Material 2

Supplementary Material 3

Supplementary Material 4

## Data Availability

The datasets or information generated and/or analysed for this paper are available within supplementary files 1 and 2.

## Abbreviations

MMR: Maternal Mortality Ratio
NMR: Neonatal Mortality Rate
SDI: Service Delivery Indicators
SARA: Service Availability and Readiness Assessment
SPA: Service Provision Assessment
DHS: Demographic and Health Survey Program
HHFA: Harmonized Health Facility Assessment
LaQshya: Labor Room Quality Improvement Initiative
NQAP: National Quality Assurance Program
NQAS: National Quality Assurance Standards
RMC: Respectful maternity care
ISQUA: International Society for Quality in Health Care
OT: Operation theatres
LRs: Labor rooms
QC: Quality Circles
RIC: Rapid Improvement Cycles
PDCA: Plan – DO – Check –Act
SMGs: State Mentoring Group DCTs = District Coaching Teams
QA: Quality Assurance
