# Supplementary material for: Appraising LaQshya’s potential in measuring quality of care for mothers and newborns: a comprehensive review of India’s Labor Room Quality Improvement Initiative
Source: BMC Pregnancy Childbirth. 2024 Apr 4;24:239. doi: 10.1186/s12884-024-06450-x (PMC10993574; doi:10.1186/s12884-024-06450-x)
Supplement: Supplementary file 1 — Supplementary Material 1 [file 12884_2024_6450_MOESM1_ESM.docx]

**Supplementary File 1 Detailed Search Strategy for PubMed Review**

|  | **Theme** | **Search Term** |
| --- | --- | --- |
| *Concept* | Quality improvement of maternal and new-born health using LaQshya Program | ("LaQshya"[All Fields] AND (((("Maternal Health"[MeSH Terms] OR "Maternal Health"[All Fields] OR "Infant Health"[MeSH Terms] OR "Infant Health"[All Fields]) AND "Or"[All Fields]) AND "Neonatal Health"[All Fields]) OR "labor, obstetric"[MeSH Terms] OR "labour"[All Fields])) AND ("quality improvement"[MeSH Terms] OR "quality assurance, health care"[MeSH Terms] OR "quality improvement*"[All Fields] OR "Quality Assurance"[All Fields] OR "Quality Monitoring"[All Fields])  AND |
| *Population* | India | (India[MeSH Terms] OR “India”)  AND |
| *Time frame* | 2017 onward | 2017/01/01:2024/02/29[dp]  AND |
| *Reporting* | English language | english [la] |
