# Supplementary material for: Appraising LaQshya’s potential in measuring quality of care for mothers and newborns: a comprehensive review of India’s Labor Room Quality Improvement Initiative
Source: BMC Pregnancy Childbirth. 2024 Apr 4;24:239. doi: 10.1186/s12884-024-06450-x (PMC10993574; doi:10.1186/s12884-024-06450-x)
Supplement: Supplementary file 4 — Supplementary Material 4 [file 12884_2024_6450_MOESM4_ESM.doc]

**Supplementary File 3: List of measures from WHO Standards for Improving Quality of Care for Mothers and Newborns not covered in the LaQshya Checklists**

|  | *Specific performance measures from WHO Standards for (QoMNC) not covered for health facility assessment under LaQshya* | | |
| --- | --- | --- | --- |
| **WHO Standards for Improving Quality of Maternal and Newborn Care (QoMNC) in health facilities** | **Input** | **Process/Output** | **Outcome** |
| **Standard 1: Every woman and newborn receive routine, evidence-based care and** **management of complications during labour, childbirth and the early postnatal period, according to WHO guidelines.** | Health facility practises and enables rooming-in to allow mothers and babies to remain together 24 h a day* | Nulliparous women with a singleton cephalic foetus at ≥ 37 weeks’ gestation undergoing caesarean section during spontaneous labour | Women who had severe post- part haemorrhage (abnormal/ any bleeding with hypotension or requiring blood transfusion) |
|  | Local arrangements to ensure that every mother knows when and where  postnatal care for herself and her newborn will be provided after discharge from the hospital | Women with third- or fourth-degree perineal tears who received antibiotics | Women who gave birth in the health facility whose uterus ruptured during labour. |
|  | Feeding of infant formula is demonstrated to mothers and family  members of newborns only when needed, with a full explanation of the hazards of improper use. | Women undergoing caesarean section according to Robson classification groups | Newborns who had birth injuries |
|  | Health staff receive in-service training.  and regular refresher sessions (every 12 months) in managing prolonged and obstructed labour. | Women whose urinalysis result was appropriately recorded during labour, childbirth, and the early postpartum period (and acted on if appropriate) | Women with caesarean section births who received a blood transfusion |
|  | Health facility does not display infant formula or bottles and teats, including on posters or placards* | Women who received any option for pain relief during labour and childbirth | Women with vaginal births who received a blood transfusion. |
|  | Health facility does not give newborns food or drink other than breast milk, unless medically indicated, and does not give pacifiers (also called “dummies” or “soothers”) to breastfeeding babies. | Newborns who were kept in skin-to-skin contact (with body and head covered) with their mothers for at least 1 h after birth. | Live preterm babies who had severe neonatal morbidity |
|  | Health facility does not promote infant formula on the wards, and samples are not distributed to mothers or staff | Women in postnatal care wards or areas in the health facility who have documented problems of blood pressure, pulse rate, vaginal bleeding, lochia or breastfeeding | Women who underwent C-section who had severe infection or sepsis after the C-section |
|  |  | Healthy mothers and newborns who received care for at least 24 h after an uncomplicated vaginal birth in a health facility. | Women who had severe systemic infection or sepsis in the postpartum period |
|  |  | Newborns on postnatal care wards or areas in the health facility for whom there is documented information on the newborn body temperature, respiratory rate, feeding behaviour and the absence or presence of danger signs | All severe neonatal morbidity due to neonatal sepsis |
|  |  | Women who gave birth in the health facility who were allowed to room-in with their newborn 24 h a day | Women who gave birth with severe systemic infection or sepsis in the postnatal period, including at readmission after delivery. |
|  |  | Women with post-partum haemorrhage in the health facility who received therapeutic uterotonic drugs | Babies born at term (≥ 37 weeks) with no major congenital malformations in the health facility who died within 7 days of birth |
|  |  | Newborns with signs of infection who received antibiotics | Low-birth-weight newborns born in the health facility who were exclusively fed on their mother’s milk during their stay in the health facility |
|  |  | Newborns of mothers with signs of infection in the health facility who received injectable antibiotics |  |
|  |  | Newborns with suspected severe bacterial infection who received appropriate antibiotic therapy |  |
|  |  | Babies born in the health facility who received early bathing and removal of the vernix within 6 h of birth. |  |
|  |  | Babies born through clear amniotic fluid in the health facility who received routine suctioning |  |
|  |  | Women in the health facility who received routine enemas at  any time before vaginal birth |  |
| **Standard 2: The health information system enables use of data to ensure early, appropriate action to improve the care of every woman and newborn.** | A birth and death registration system in place in health facility linked to the national vital registration system | Perinatal deaths occurring in the health facility reviewed with standard audit tools |  |
|  | System for classifying diseases and birth outcomes, including death, aligned with the ICD |  |  |
| **Standard 3: Every woman and newborn with condition(s) that cannot be dealt with effectively with the available resources is appropriately referred.** |  |  | Newborns referred from the facility who reached the referral facility without hypothermia |
| **Standard 4: Communication with women and their families is effective and responds to their needs and preferences.** | A written, up-to-date policy with clear goals, operational plans and monitoring mechanisms on interpersonal communication and counselling skills of health care staff | Women who reported that they were given the opportunity to discuss their concerns and preferences | Women who reported that their needs and preferences were taken into account during labour, childbirth and postnatal care |
|  | Supportive supervision of staff in interpersonal communication, counselling and cultural competence every three months | Women who were satisfied that the facility met their religious and cultural needs |  |
| **Standard 5: Women and newborns receive care with respect and preservation of their dignity.** | System for mothers of small, sick newborns to be close to nurse their babies | Women who were aware of the existence and location of a complaints box | Women who were satisfied with the degree of privacy during their stay in the labour and childbirth areas. |
|  | Written accountability mechanisms for redress in the event of mistreatment | Proportion of complaints received about respect and preservation of the dignity of women and their families | Women who felt they had shared decisions about their labour, birth, and postnatal care. |
|  | Written, up-to-date policy and protocols that outline women’s and families’ right to make a complaint about the care received and has an easily accessible mechanism to hand over complaints | Women who were aware that they had the right to accept or refuse treatment | Women who made a complaint whose complaints were acted upon without repercussions |
|  | Written accountability mechanisms for redress if women are denied informed choice, and the mechanism is displayed | Women who were aware that they had the right to accept or refuse treatment | Women who reported having been treated with respect and their dignity preserved |
|  | Written accountability mechanisms for redress if women are denied informed choice, and the mechanism is displayed |  |  |
| **Standard 6: Every woman and her family are provided with emotional support that is sensitive to their needs and strengthens the woman’s capability.** | Staff orientation in nonpharmacological and pharmacological pain relief and in-service training or sessions at least once in the preceding 12 months | Women who gave birth in the labour position of their choice | Women who would recommend childbirth in that facility. |
|  | Written, up-to-date protocol, which is explained to women and their families,  to minimize unnecessary interventions, support normal labour and strengthen the woman’s capability,  so that she feels in control of her childbirth experience | Women facing adverse outcome who received additional emotional support from health facility staff |  |
|  |  | Women who were ambulatory during the first stage of labour |  |
| **Standard 7: For every woman and newborn, competent, motivated staff are consistently available to provide routine care and manage complications** | Standard procedures for recruitment, deployment, motivation and retention of all staff | Staff oriented to their functions, roles and responsibilities in the facility or unit to which they are assigned | Staff at the health facility who reported being “highly satisfied” with their job. |
|  |  | Skilled birth staff at the health facility who received a written job description on deployment to the facility | Saff who could identify and report on at least one clinical improvement activity in which they were personally involved in the past 6 months. |
|  | Standard procedures and plans for recruitment, deployment, motivation (recognition and reward scheme) and retention of all staff | Staff assessment at least once in the preceding 12 months | Staff at the health facility who were actively considering looking for a new job. |
|  | Sufficient numbers of educated, competent, licensed, motivated, regulated skilled birth attendants with an appropriate skills mix, working in multidisciplinary teams | Monthly team meetings to review competence and quality improvement activities |  |
|  | Supportive environment for staff development, with regular supportive supervision | Monthly interactions with mentors for clinical competence and improve performance. |  |
|  | Inter-professional collaborative practice, with clear roles and responsibilities based on the professional scope of practice and care needs | Staff engagement in at least two quality improvement team meetings and participation in quality improvement activities in the last six months |  |
|  | Written, up-to-date leadership structure, with defined roles and responsibilities and lines of accountability for reporting | Number of supervisory visits to support clinical competence and performance improvement (in  the past three months) |  |
|  | One monthly meeting to review data, monitor quality improvement performance, make recommendations to address any identified problems, honour those who have performed well and encourage staff who are struggling to improve |  |  |
|  | At least two annual meetings with stakeholders (e.g. the community, service users, partners) to review its performance, identify problems and make recommendations for joint actions to improve quality |  |  |
|  | Leaders trained in quality improvement and leading change (use of information, enabling behaviour, continuous learning) |  |  |
|  |  |  |  |
|  | Leaders trained in leadership and management skills |  |  |
|  | Policy for staff to provide feedback to the facility management on quality improvement and their performance |  |  |
| **Standard 8: The health facility has an appropriate physical environment, with adequate water, sanitation and energy supplies, medicines, supplies and equipment for routine maternal and newborn care and management of complications.** | Functioning hand hygiene station per 10 beds, with soap and water or alcohol-based hand rubs, in all wards |  |  |
|  | Energy management plan with adequate budget, maintained and regulated |  | Women who had severe pre-eclampsia or eclampsia in the health facility who did not receive the full dose of magnesium sulphate because of a stock-out. |
|  | Preventive risk plan to manage and improve water, sanitation and hygiene services, including for infection prevention and control |  | Nulliparous women with a singleton cephalic foetus at > 37 weeks of gestation who underwent a C-section during spontaneous labour. |
|  | Fuel management plan and a local buffer stock, with budget for vehicles, cooking and heating requirements |  |  |
|  | A dedicated ward for admitting sick and unstable small babies |  |  |
|  | Facilities for rooming-in for all women and babies to remain together 24 h a day |  |  |
|  | Functional diagnostic ultrasound machine and trained health staff to conduct a basic obstetric ultrasound examination |  |  |
|  | Dedicated funds for essential medicines and supplies |  |  |
